# Homelessness in the district of Nipissing of Ontario, Canada before, at the onset and during the COVID-19 pandemic: a trend analysis (2018–2021)

**DOI:** 10.1186/s12889-023-16314-2

**Published:** 2023-07-19

**Authors:** Megan Odd, Amir Erfani

**Affiliations:** 1 Centre of Access, Interdisciplinary Studies and Lifelong Learning, Canadore College of Applied Arts and Technology, 100 College Drive, North Bay, ON P1B 8K9 Canada; 2 Department of Sociology , Nipissing University, 100 College Drive, Box 5002, North Bay, ON P1B 8L7 Canada

**Keywords:** Homeless, Homelessness, COVID-19, Pandemic, Canada, Poverty

## Abstract

**Background:**

Homelessness is a growing social concern experienced across Canada. In Ontario, specifically in the District of Nipissing, the issue has become larger with an increasing number of homeless individuals. Previous research has described the demographic composition of the homeless population both in the Nipissing District of Ontario and in the city of North Bay. However, no studies have examined homelessness in this region before, at the beginning and during the COVID-19 pandemic. This research investigates structural and individual-level barriers and factors that are associated with becoming homeless or remaining homeless.

**Methods:**

This study utilizes data from the 2018 (n = 147), 2020 (n = 254), and 2021 (n = 207) homelessness enumeration surveys, conducted in the District of Nipissing, Ontario by the District of Nipissing Social Services Administration Board. This study employs quantitative, descriptive analyses to examine trends and socio-demographic variations in the reasons of homelessness, barriers to housing, episodic and chronic homelessness before, at the beginning, and during the COVID-19 pandemic.

**Results:**

The results revealed a rise in the proportion of male homeless (57% vs. 64%), and first-time homelessness among those aged 35–44 (3%, vs. 15%) and 55–64 (1% vs. 5%) at the onset and during the pandemic. The sleep location of homeless individuals was also influenced by the pandemic, where emergency shelter use dropped to half during 2020–2021(33% vs. 17%), while the use of locations (hotel/motels) where proper pandemic protocols and social distancing were possible increased sharply from 2 to 12% of homeless individuals. With the onset of the pandemic, chronic homelessness and one-episodic homelessness increased, suggesting that individuals are becoming homeless and staying homeless for prolonged periods. The barriers to housing during the pandemic were largely addiction, substance use and the inaccessibility of safe and secure rental units, while the corresponding barriers before the pandemic were mainly low income.

**Conclusions:**

The rise in male homelessness, age at first-time homelessness and interpersonal conflict causing homelessness at the onset and during the pandemic suggest that policy makers need to focus on providing homeless supports to these groups of homeless populations at the time of pandemic.

**Supplementary Information:**

The online version contains supplementary material available at 10.1186/s12889-023-16314-2.

## Background

Homelessness refers to the situation in which an individual has no stable, permanent, and appropriate housing, or an immediate prospect of [3]. Over the last few decades, the prevalence of individuals experiencing homelessness in Canada has been increasing [1, 2]. Though this trend could be the result of the evolving definition [1, 3], or the changing pathways into homelessness, the recent pandemic of COVID-19 has made it an increasingly pressing issue in Canada, especially in the northern communities such as the District of Nipissing in Northeastern Ontario.

During the 1960’s and 1970’s, the federal government “invested heavily in adequate housing for Canadians” [4]. To further accommodate the lack of housing, the *National Housing Act* was amended in 1973 allowing 20,000 housing units to be built each year following [4]. However, when the 1980’s approached, these funds were withdrawn [5, 6]. This event has been documented as the point-in-time where the rise of modern mass homelessness in Canada began. To combat the housing crisis, the federal government introduced a new cabinet position in 1999 titled the “Minister Responsible for Homelessness” [7] and reallocated over $600 million dollars under the *National Homelessness Initiative* (NHI), which would take place from 1999 to 2003. Although a notable effort, the need for adequate housing had since exceeded the available funds and was further highlighted as a key catalyst for widespread homelessness in Canada [8].

Nationally, the national average number of individuals experiencing homelessness in Canada was declared at more than 235,000 – approximately 30,000 on any given night within the calendar year in 2021 [1]. These figures represent an overall increase in homelessness across Canada from both 2016 and 2018 [2, 9]. As a result, the need for shelter space increased. Statistics Canada examined fluctuations in the number of homeless shelters and shelter beds across the nation [10]. They found that between the years 2016 and 2019, the overall number of both homeless shelters (including emergency shelters, transitional housing and violence against women shelters), and the beds within them increased.

Provincially, the number of homeless individuals in Ontario, where the Nipissing District is located, increased from 4,203 homeless in 2010 to 9,350 individuals in 2017, and males made up 71% of homeless population in 2017 [1]. However, in the North-East regions of Ontario, female individuals made up a larger proportion (53%) of the homeless population. Moreover, homeless individuals with Indigenous ancestry were overrepresented, those between the ages of 20 and 59 comprised a large portion (44.1%), and that physical and mental health problems were cited as a main contributor [11]. A second study conducted by Kauppi in 2015 in Sudbury, Ontario, found results counter to that of the Cochrane study. Here, males drastically outnumbered (53.9%) females (32.6%). A more recent study from Thunder Bay, Ontario found comparable results to that of Sudbury, where the total homeless population was comprised of more males (69.7%) than females (29.6%) [12]. The most frequent responses when asked what the main reason for homelessness was in Northern Ontario communities include physical and mental health problems, substance use, inability to pay rent, and interpersonal or spousal conflict [11, 13, 14].

At the community level, homeless population in the city of North Bay, located in the Nipissing District, had a gender composition evenly split between males (49.2%) and females (50.8%), and mostly fell between the ages of 20 and 49 (45.7%) [15]. Like other findings in surrounding communities, Indigenous individuals were overrepresented at 26% of the total population experiencing homelessness, while they comprise approximately 7.9% of the city’s population [16]. The most documented reasons for homelessness in North Bay were unemployment or lack of income, problems with getting and maintaining social assistance, physical and mental health issues, and family problems including domestic violence.

Studies showed that during the pandemic, homeless individuals’ access to social and healthcare resources have declined, their diseases and infections have increased [17], and they were more likely to test positive for COVID-19 compared to those not experiencing homelessness [18]. Beyond this evidence, there is little information on the prevalence of homelessness and characteristics of the homeless population before, at the onset and during the pandemic. A comparative analysis would provide insight on the role of the pandemic on the incidence of homelessness and changing sociodemographic characteristics of homeless individuals. With the availability of recent data during these three periods, this study aims to address the gap by studying the number of homeless individuals, the factors related to susceptibility, and the population profile of homelessness in the Nipissing District before, at the start, and during the COVID-19 pandemic using data from the 2018, 2020, and 2021 homeless enumerations. The results of this study will inform agencies and stakeholders providing services to homeless population and marginalized and vulnerable individuals.

The literature indicates that the demographics and factors related to homelessness differ by geographic locations, prompting the need for a district-specific study. Therefore, the current research aims to study the number of homeless individuals, their demographic profiles, and the barriers that directly prevent them to obtain and secure housing before, at the start and during the COVID-19 pandemic in the Nipissing District of Ontario.

## Methods

### Data

This study utilized the data obtained from three District-wide enumerations of homeless individuals conducted in 2018, 2020, and 2021 by three face-to-face “enumeration” surveys. The surveys were conducted among individuals experiencing homelessness across the communities located in the District of Nipissing, Ontario, Canada. These data are observational and have been captured in real-time.

The surveys were funded by and administered (face-to-face) by the District of Nipissing Social Services Administration Board (DNSSAB) and associated homeless service and support agencies, which helped the successful execution of the enumerations in the District’s communities [19]. The completed survey questionnaires were returned to the project’s head analyst for reconciliation and de-duplication. Microsoft Excel files containing the survey results were then created. After removing the identifying information of homeless individuals, the DNSSAB granted access to the survey data files for this study. Once the files were provided to the principal researcher, they were transformed to be compatible with the SPSS software for analysis. This study has received a Nipissing University ethic approval (File number: 102,855).

### Methods of enumeration

Point-in-Time (PiT) count was the enumeration method used by the District of Nipissing Social Services Administration Board (DNSSAB) and associated community partners to conduct the most recent homeless counts in the Nipissing District. PiT counts have been considered one of the best methods, or “gold standard” for homelessness enumerations by academics [25]. Point-in-Time counts consisted of a 24-hour enumeration period, during which volunteers and service providers completed a physical count of those experiencing homelessness in the Nipissing District. On October 13, 2021, the count teams visited homeless hot spots in all the municipalities located in the Nipissing District, namely North Bay, Mattawa, West Nipissing, Temagami, East Ferris, Chisholm, and South Algonquin, and identified and counted individuals experiencing homelessness. These individuals were then asked if they would be willing to participate in the enumeration survey, answering survey questions. In the three 2018, 2020, and 2021 enumerations, 182, 293, and 300 homeless individuals were counted respectively, out of which 147, 254, and 207 homeless persons participated in the surveys, yielding 80.7%, 86.6%, and 69.0% response rates respectively. The survey questionnaires collected data about demographic characteristics of homeless individuals, their history and reasons of homelessness, sleep location, and barriers to housing in the Nipissing District.

### Sociodemographic and homelessness characteristics

The variables used in this study include age of respondent, their gender identity, Indigenous identity, their experience (if any) in the foster care system and their current source of income. The surveys also asked respondents for the age at which they experienced homelessness for the first time. Questions specific to the number of times each respondent had experienced a homeless period over the last 12 months (episodic homelessness) and the cumulative amount of time these periods lasted (chronic homelessness) were also asked. To determine the main reasons for homelessness in the Nipissing District, respondents were asked to indicate the main reason(s) behind their most recent housing loss. To go a step further, respondents were then asked what the main barriers to housing they were currently facing, that is, what problems are currently preventing them from securing and maintaining housing. The Government of Ontario selected these data points for the purposes of comparison across Districts in the province. The identical questions used in the three enumeration surveys allowed examining changes in homelessness over time (see Appendix A for a sample of questionnaire).

### Statistical analysis

The survey data was analyzed to describe changes in the homelessness before, at the onset and during the COVID-19 pandemic. The data from the three enumerations were compared to uncover any possible changes in the overall homeless population in the Nipissing District, which can be attributed to the pandemic. This analysis also allowed us to compare the distribution of homelessness among various demographic sub-populations over time, as well as examine the fluctuations in the factors associated with experiencing homelessness. In addition to percentage distribution, Chi-square test was used to examine whether the differences in the homeless individuals’ characteristics over the three survey years were statistically significant or not at p-value ≤ 0.05. SPSS 26.0 was used to analyze data.

## Results

**Fig. 1:**
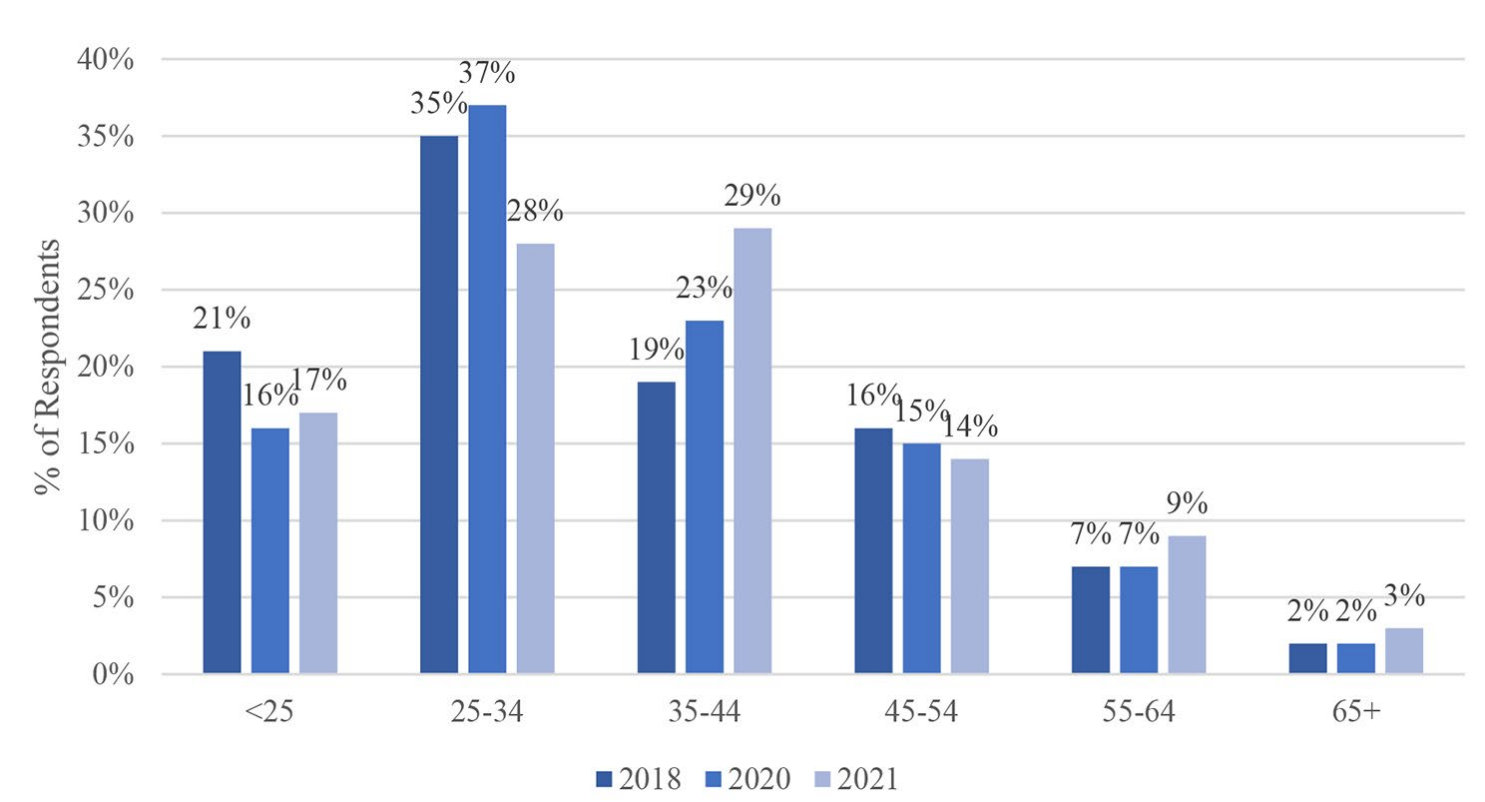
Age of Respondent

**Fig. 2:**
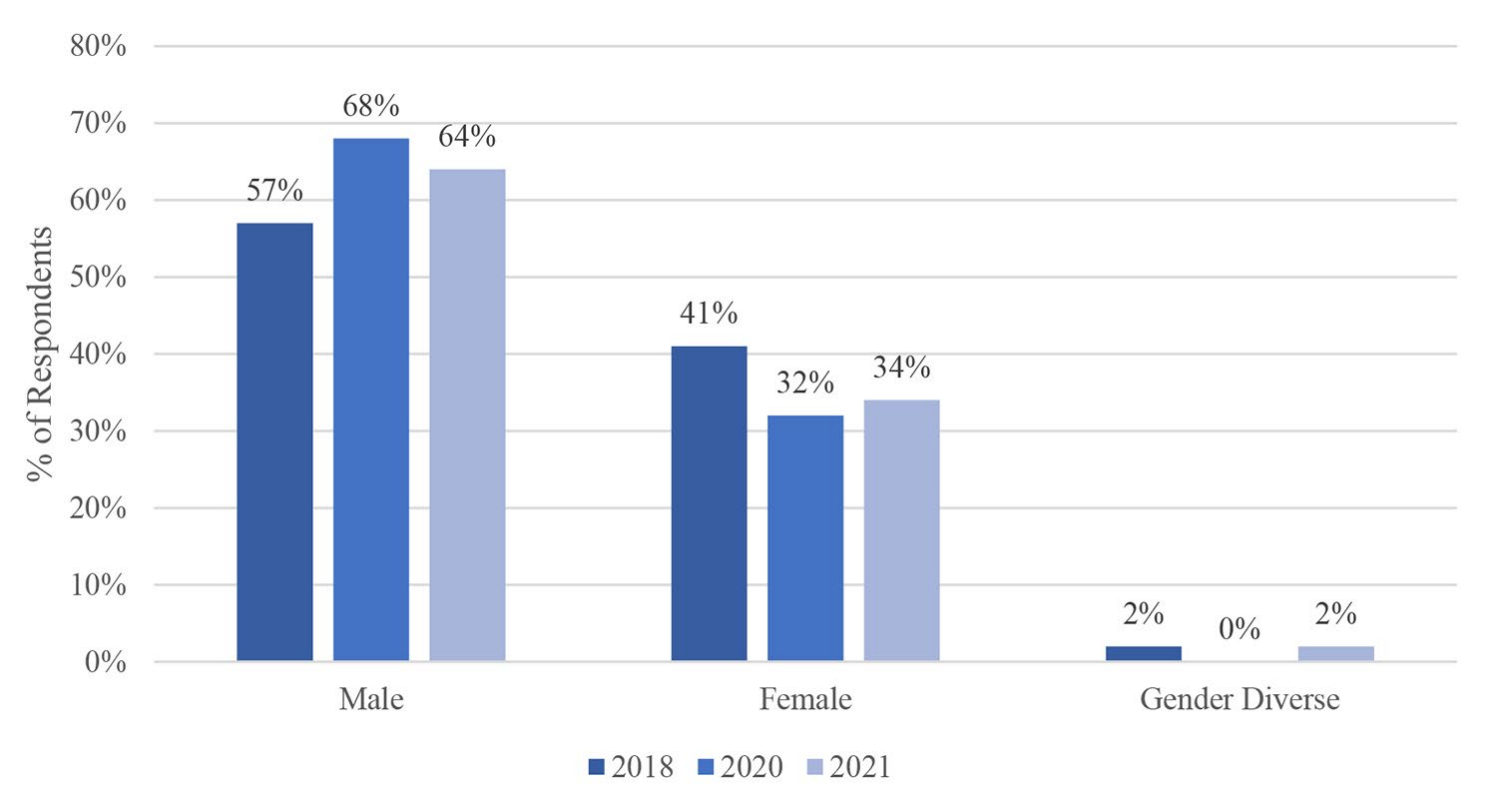
Gender Identity of Respondent

**Fig. 3:**
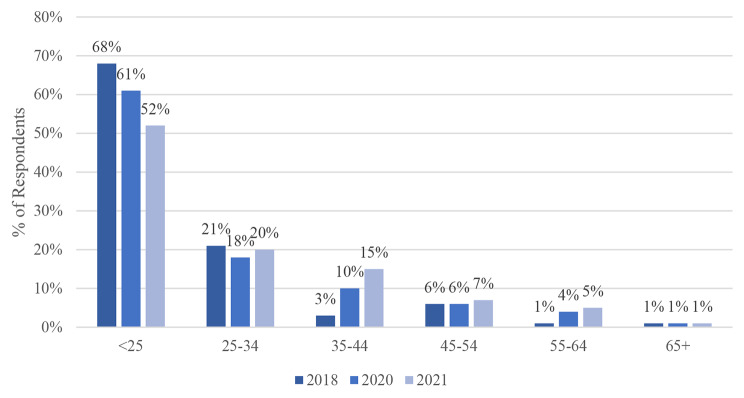
Age of Respondent at First Homelessness

**Fig. 4:**
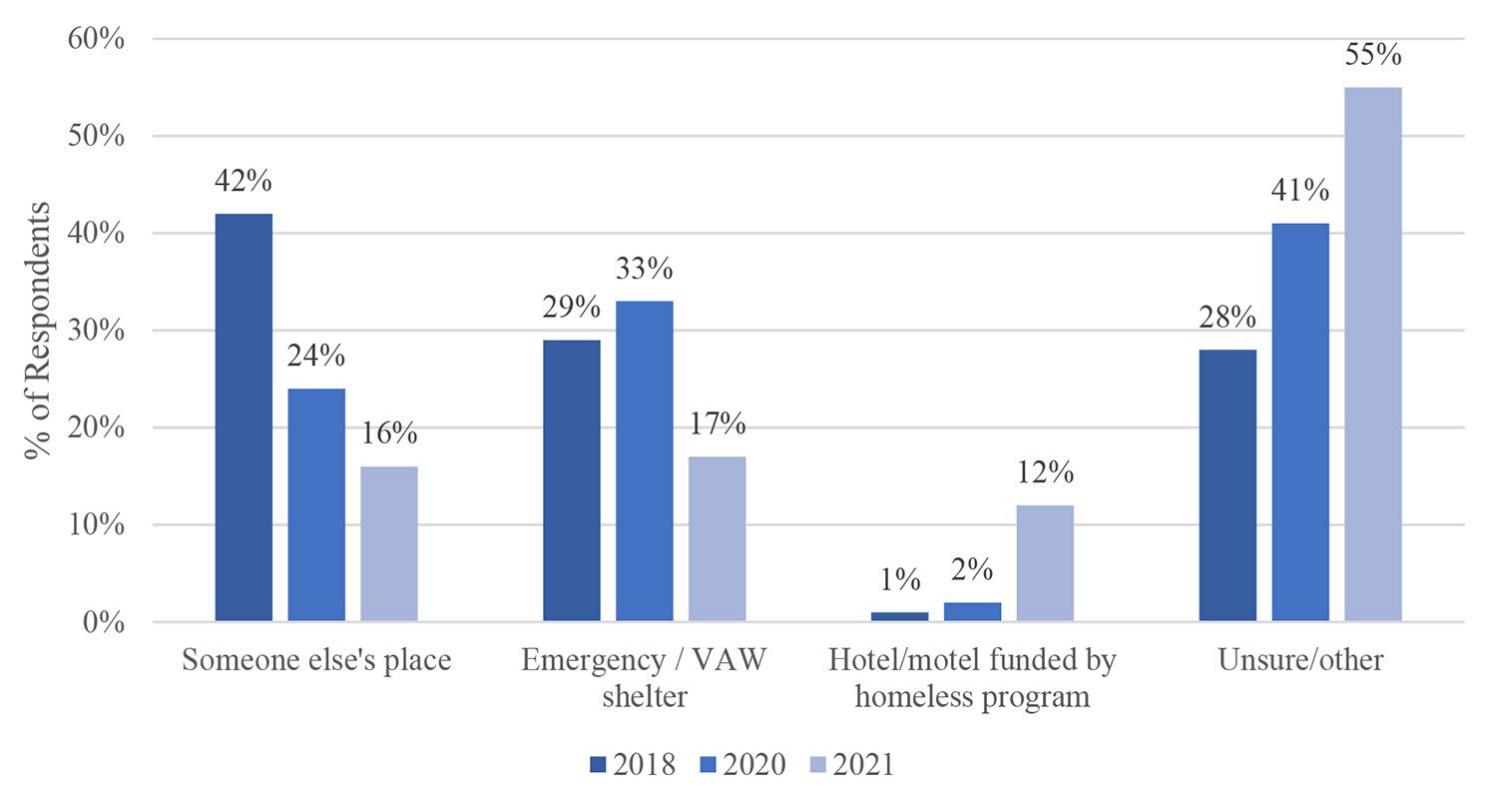
Respondent Sleeping Location

**Fig. 5:**
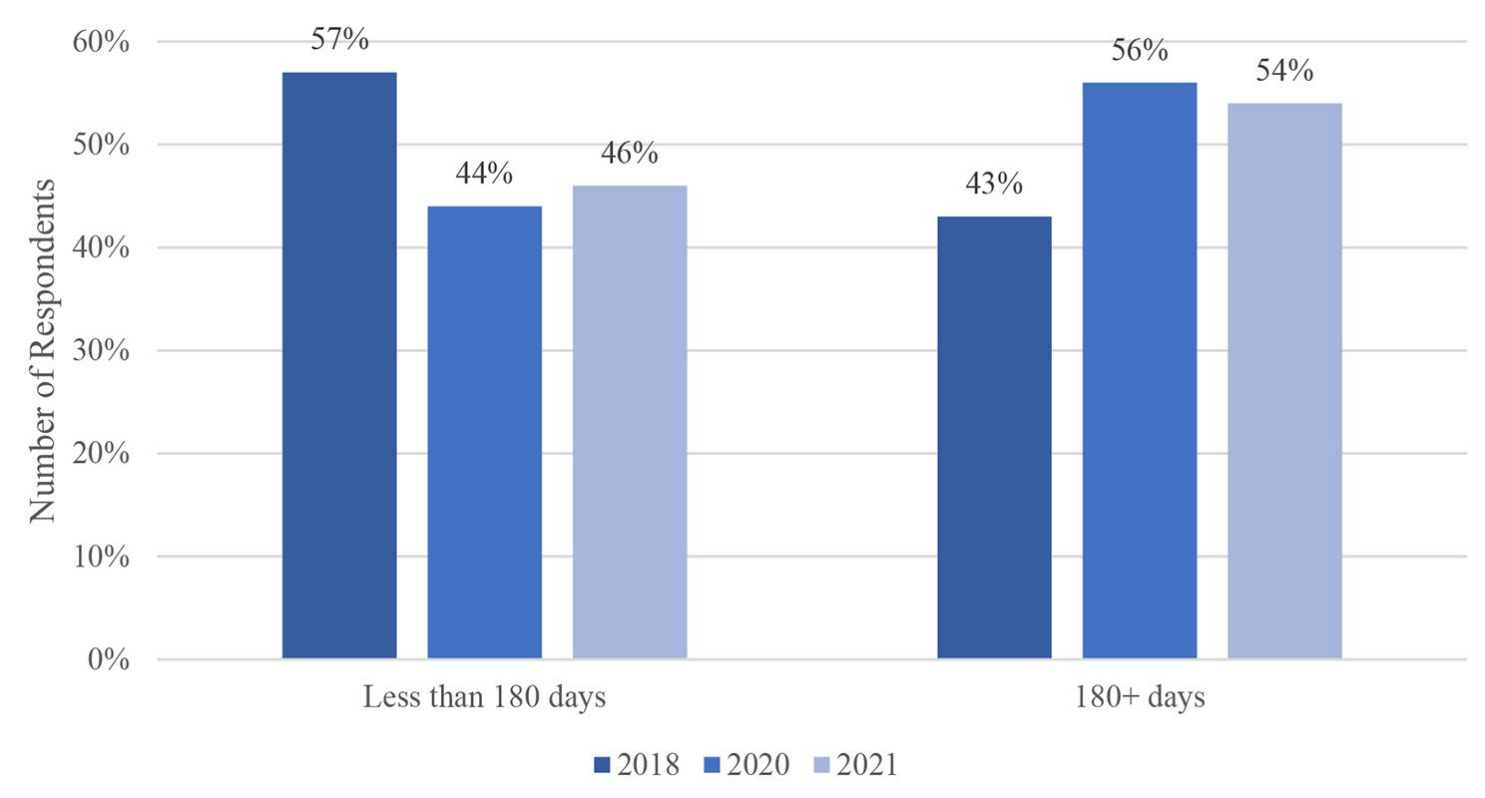
Cumulative Homelessness

**Fig. 6:**
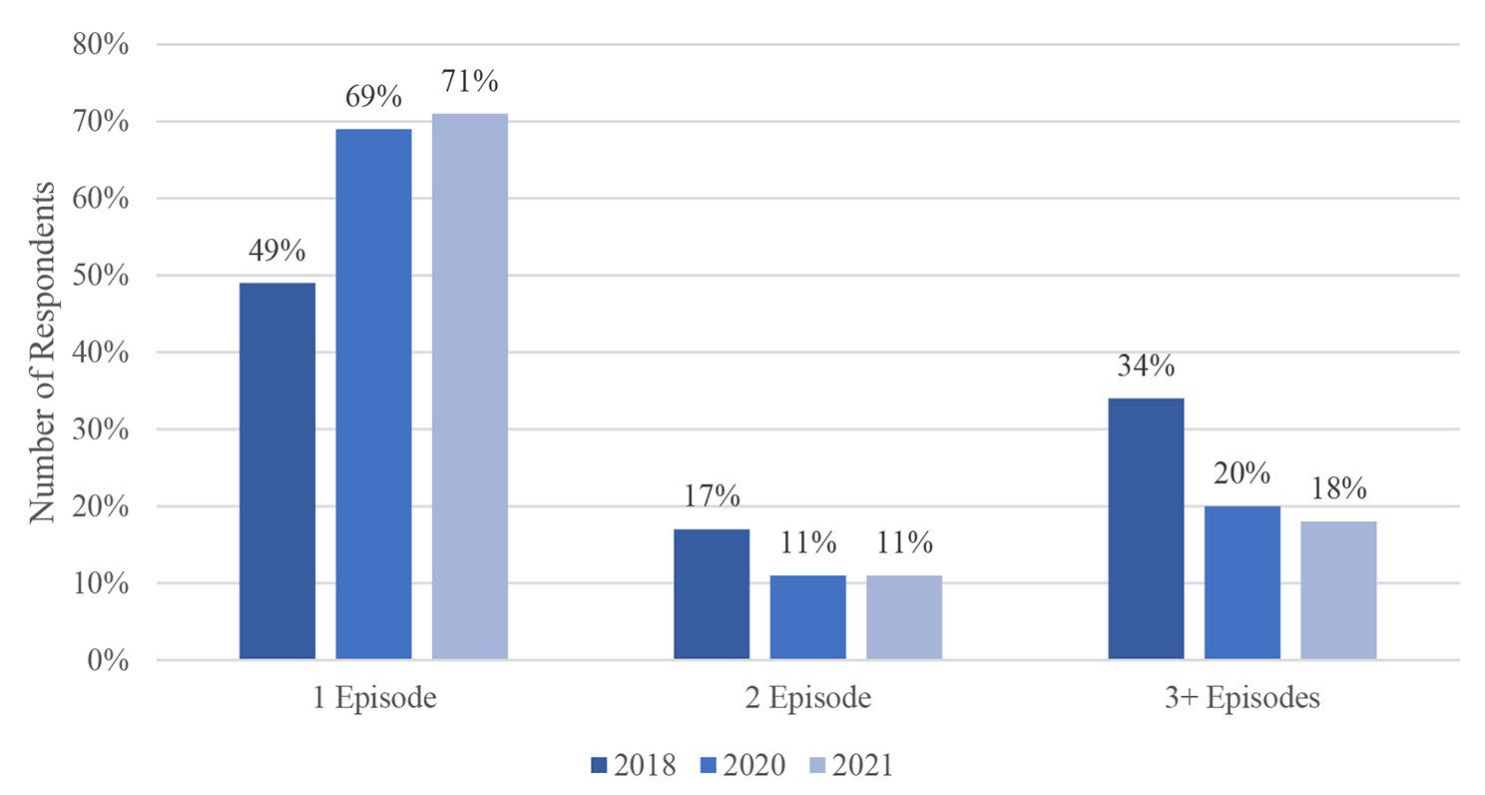
Episodic Homelessness

**Fig. 7:**
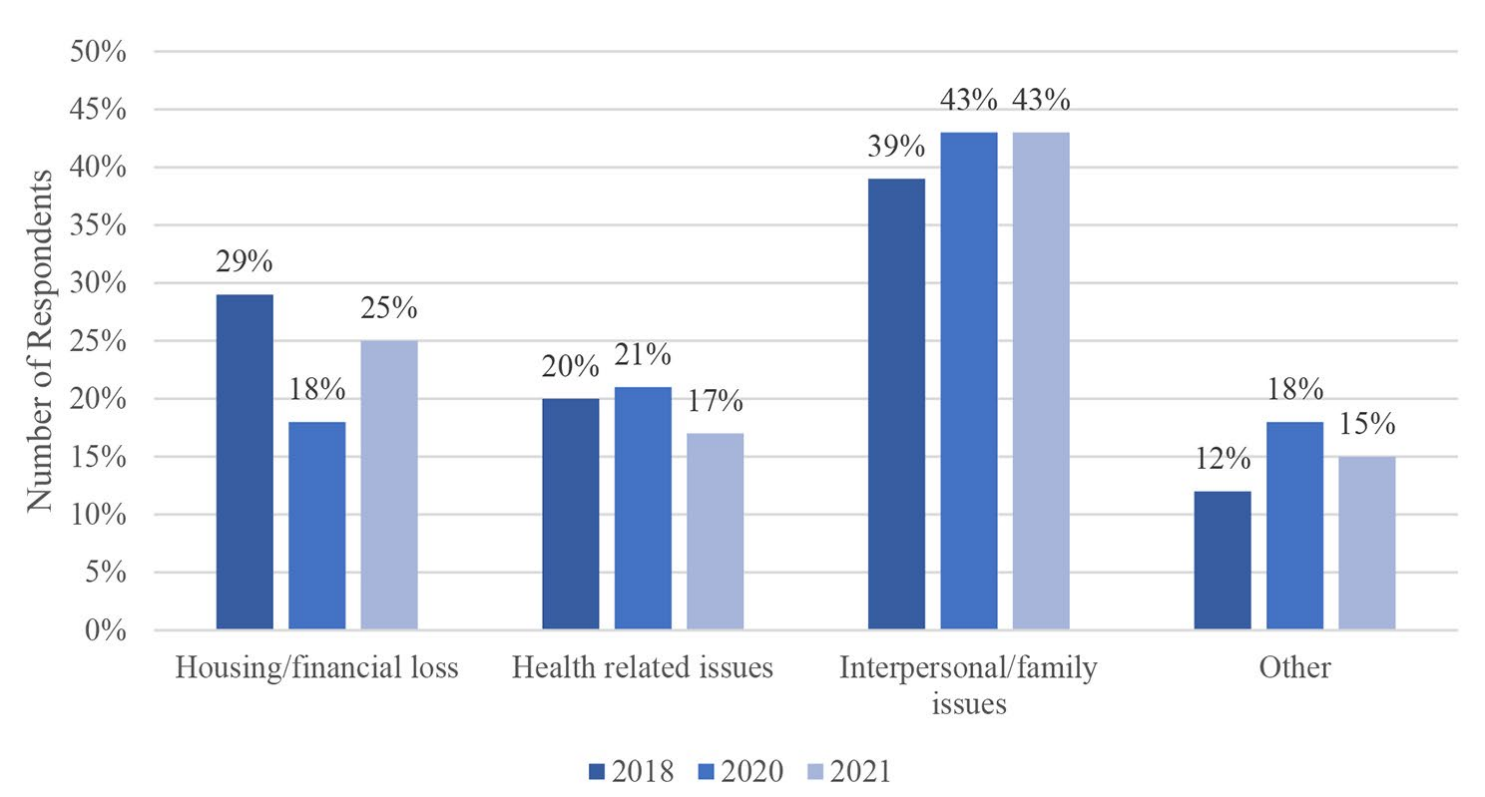
Respondents’ Most Recent Reason for Housing Loss

**Fig. 8:**
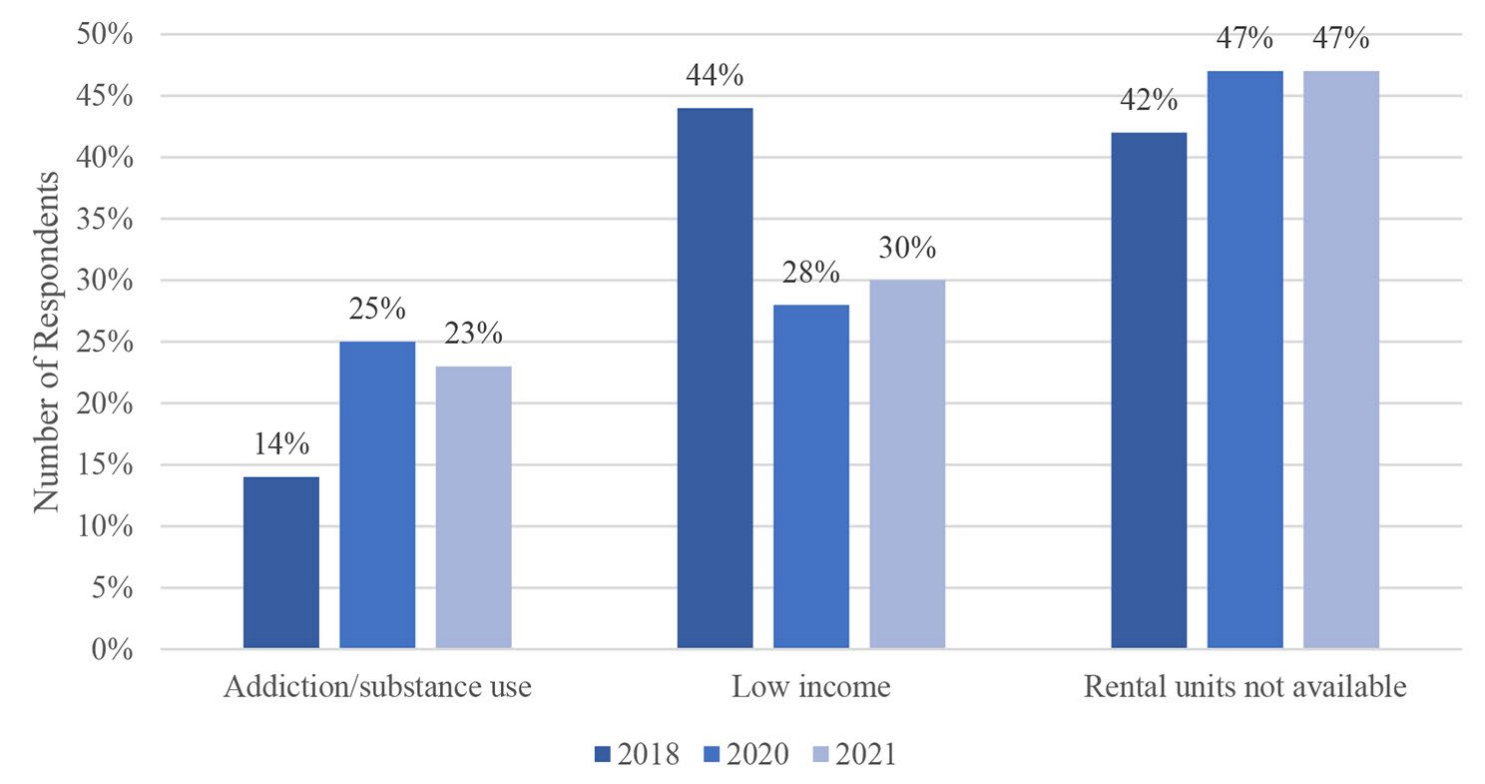
Respondents’ Current Barriers to Housing

Table 1 shows the distribution of homeless individuals across the three surveys according to their sociodemographic and homeless characteristics. The results indicate significant differentials in the homeless characteristics of individuals before, at the onset and during the COVID-19 pandemic (2018, 2020, and 2021). However, their age, gender and Indigenous status did not differ significantly across the three periods. The differences in these characteristics have been illustrated and described in Figs. 1, 2, 3, 4, 5, 6, 7 and 8.

**Table 1:** Frequency and percent distributions of homeless individuals enumerated in the Nipissing District Homeless Enumeration Surveys, 2018–2021, by *selected* characteristics

Characteristic	2018 (n = 147)	2020 (n = 254)	2021 (n = 207)
	n (%)	n (%)	n (%)
**Age of respondent** < 25 25–34 35–44 45–54 55–64 65+ Total*	31 (21.1) 51 (34.7) 28 (19.0) 24 (16.3) 10 (6.9) 3 (2.0) 147 (100.0)	40 (16.0) 92 (36.8) 58 (23.2) 36 (14.4) 18 (7.2) 6 (2.4) 250 (100.0)	35 (16.9) 56 (27.1) 61 (29.5) 30 (14.5) 19 (9.2) 6 (2.8) 207 (100.0)
Chi-Square (p-value) = 10.3 (0.42)			
**Gender ID of respondent** Male Female Gender diverse Total*	84 (57.1) 60 (40.8) 3 (2.1) 147 (100.0)	170 (67.7) 81 (32.2) 0 (0.0) 251 (100.0)	130 (64.4) 68 (33.6) 4 (2.0) 202 (100.0)
Chi-Square (p-value) = 8.7 (0.07)			
**Indigenous ID of respondent** Indigenous Non-Indigenous Total*	65 (45.5) 78 (54.5) 143 (100.0)	98 (41.8) 136 (58.2) 234 (100.0)	92 (44.6) 114 (55.4) 206 (100.0)
Chi-Square (p-value) **=** 0.57 (0.75)			
**Age at first homelessness** < 25 25–34 35–44 45–54 55–64 65+ Total*	98 (68.1) 29 (20.1) 4 (2.7) 9 (6.3) 2 (1.4) 2 (1.4) 144 (100.0)	147 (60.5) 44 (18.1) 25 (10.3) 15 (6.2) 9 (3.7) 3 (1.2) 243 (100.0)	101 (51.5) 40 (20.4) 29 (14.8) 13 (6.6) 10 (5.1) 3 (1.5) 196 (100.0)
Chi-Square (p-value) **=** 19.7 (0.03)			
**Cumulative homelessness** 1-179 days 180 + days Total*	75 (56.8) 57 (43.2) 132 (100.0)	100 (44.1) 127 (55.9) 227 (100.0)	85 (45.7) 101 (54.3) 186 (100.0)
Chi-Square (p-value) **=** 5.9 (0.05)			
**Episodic homelessness** 1 episode 2 episodes 3 + episodes Total*	66 (49.6) 22 (16.5) 45 (33.8) 133 (100.0)	158 (69.3) 24 (10.5) 46 (20.2) 228 (100.0)	132 (70.9) 21 (11.4) 33 (17.7) 186 (100.0)
Chi-Square (p-value) = 49.4 (0.0001)			
**Where are you staying tonight?** Someone else’s place Emergency shelter Hotel/motel funded by homeless program Unsure/other Total*			
	61 (41.8) 43 (29.4) 2 (1.4) 40 (27.4) 146 (100.0)	62 (24.4) 82 (32.2) 6 (2.5) 104 (40.9) 254 (100.0)	33 (16.0) 35 (17.0) 24 (11.7) 114 (55.3) 206 (100.0)
Chi-Square (p-value) = 73.18 (0.0001)			
**Reason for housing loss** Housing/financial loss^1^ Health related issues^2^ Interpersonal/family issues^3^ Other^4^ Total**	67 (29.3) 46 (20.1) 88 (38.4) 28 (12.2) 229 (100.0)	67 (18.2) 77 (20.8) 157 (42.6) 68 (18.4) 369 (100.0)	76 (25.3) 52 (17.3) 129 (42.8) 44 (14.6) 301 (100.0)
Chi-Square (p-value) = 13.8 (0.03)			
**Barriers to housing** Addiction/substance use Low income Rental units not available Total**	27 (13.6) 88 (44.2) 84 (42.2) 199 (100.0)	133 (25.3) 146 (27.9) 245 (46.8) 524 (100.0)	108 (23.4) 108 (29.8) 207 (46.8) 423 (100.0)
Chi-Square (p-value) = 28.3 (0.0001)			

^1^ includes Building Sold or Renovated [13], Complaint [3], Left Community [10], Not Enough Income for Housing [24], Owner Moved In [3], and Unfit/Unsafe Housing [23]

^2^ includes Hospitalization or Treatment Program [5], Mental Health [14], Physical Health [4], and Substance Abuse [29]

^3^ includes Experienced discrimination [3], Conflict with tenant/landlord [21], Conflict with parent/guardian [14], Conflict with spouse/partner [28], Conflict with other [18], Death of family member [5], Departure of family member [1], Experienced abuse from parent/guardian [7], Experienced abuse from spouse/partner [15], Experienced abuse from other [4], and Family breakdown [13]

^4^ includes Related to foster care [3], Incarceration [25], Other [16]

* The discrepancies between the values of Total and the total number of cases in each survey are due to excluding “missing” and “don’t know” values from the analyses. So, percentages are based on the “valid” cases

** Total for these two variables refer to the total number of “responses”, which are greater than the total number of enumerated persons, because respondents were allowed to give more than one response. So, the percentages are based on “responses”

### Age

Figure 1 shows that, in 2018, the largest proportion of respondents fell between 25 and 34 years of age (35%). This trend continued in 2020, with 37% of survey respondents in this age group. However, during the pandemic (2021), homeless individuals were predominantly aged 35–44 (29%). Further, the number of respondents aged 55–64 and 65 + slightly increased over the three years. Age of respondents was not significantly different before, at the onset and during the pandemic (Table 1, p = 0.42).

### Gender

The overall dispersion of gender among homeless individuals has remained static over the three enumerations, where males represent the largest proportion compared to females or gender diverse individuals. This dispersion, however, has not always been of equal proportion. In 2018, males represented 57% of the total homeless population, followed by female and gender diverse individuals with 41% and 2% respectively. This gender inequity has increased with the pandemic, where 68% of homeless individuals were male and the rest (32%) female. This trend carried into 2021, where males (64%) continued to out-number females (34%). There was also a notable increase in the number of homeless gender diverse individuals (2%) during the pandemic (Table 1).

### Indigenous status

Indigenous individuals have represented roughly the same percentage of the total homeless population before (45.5%), at the onset (41.8), and during (44.6%) the pandemic (Table 1). Considering Indigenous peoples make up roughly 4.3% of the country’s total population, and approximately 14% of the Nipissing District’s population [10], the 42–45% representations in this study suggests high overrepresentation. The difference in Indigenous status was not statistically significant (p = 0.75).

### Age at first homelessness

The age at which respondents experienced first-time homelessness has increased with the pandemic. Those who became homeless for the first time before the age of 25 dropped from 68% to 2018 to 61% and 52% in 2020 and 2021 (Table 1; Fig. 3). However, those who experienced first-time homelessness between 35 and 44 increased from 6% to 2018 to 10% and 15% in 2020 and 2021, respectively. Similarly, the proportion of those between 55 and 64 also increased.

### Current sleeping location

It is apparent more recently that fewer individuals experiencing homelessness can stay with someone they know (Fig. 4). In 2018, 42% of respondents indicated that they were staying at someone else’s place. However, this decreased to 24% and 16% in 2020 and 2021. While the use of emergency shelters dropped from 33 to 17% during 2020–2021, the use of hotels/motels funded by homelessness programs increased by six times (from 2 to 12%) in the same period. The proportion of homeless individuals who were uncertain of their sleeping location rose during the pandemic (from 28% to 2018 to 41% and 55% in 2021). The differences in sleeping location across the three survey years were statistically significant (p = 0.05).

### Cumulative homelessness

The cumulative length of time that an individual has spent homeless over the last three enumerations in the District of Nipissing has increased (Fig. 5). In 2018, a larger proportion of respondents spent 179 days or less homeless (57%), compared to those who spent 180 days or more (43%). This changed at the beginning of the pandemic, where we saw a decrease in respondents spending less than six months homeless (44%) compared to the 56% who were homeless for six months or more. This trend continued into 2021. Differences in chronic homelessness during 2018–2021 are statistically significant (p = 0.05).

### Episodic homelessness

The number of times that an individual experienced a homeless episode has decreased. In 2018, 49% of respondents indicated that they had experienced homelessness once during the last 365 days. This increased to 69% and 71% in 2020 and 2021 respectively. However, the number of those who experienced a homeless episode twice, three, or more than three times has decreased. That is, individuals experienced fewer homeless episodes during the pandemic. The differences in ‘Episodic Homelessness’ are statistically significant (p = 0.0001).

### Reason for housing loss

Results in Fig. 7 show that across all three surveys, the most common reason for housing loss was *Interpersonal/Family issues* (39% in 2018 vs. 43% in 2020 and 2021). The results also show a u-shape pattern in the proportion of those reporting *Housing/Financial Loss* as the reason for housing loss, decreasing from 29% to 2018 to 18% in 2020 and then increasing to 25% in 2021. A decrease, however, was seen in health-related issues. These variations are statistically significant (p = 0.032).

### The barriers to housing

As a barrier to housing, addiction and substance use have increased drastically since the onset of COVID-19, from 14% to 2018 to 25% in 2020. In 2018, “low income” was the leading barrier to housing in the Nipissing District. However, the trends show a decrease from 44% to 2018 to 30% in 2021. Respondents from 2020 to 2021 indicated that the inaccessibility of rental units was the main barrier they faced (46% and 47%, respectively). “Other” barriers to housing have also increased. This includes pets, children, personal choice, stability, parole, landlord issues, need for extra support, issues with transportation, no identification, no computer of cell phone, and lack of references. The differences in the barriers over the three survey years are statistically significant (p = 0.001).

## Discussion

The results of this study identified patterns that highlight key similarities and changes in homelessness in the Nipissing District. The age pattern of homeless population showed that the proportion of local homeless population in the middle and upper age groups have been slightly increasing over the three periods (though not being significant), suggesting that those experiencing homelessness may have continued to live in the District over time. Urban areas often offer greater resources than rural for homeless individuals, including services and supports. Taylor [20] wrote that homelessness services and supports are commonly scarce in rural communities, and often do not provide a “culturally safe space” for the Indigenous homeless population. In the District of Nipissing specifically, most services and supports are located in the city of North Bay, Ontario. Urban city-centres also provide the convenience of public transportation that individuals experiencing homelessness can use to access resources in a timely manner. A particularly interesting reason why the homeless may continue to live in urban areas is to foster social ties and build friendships. Those with weak social ties are often more susceptible to homelessness [21]. These individuals may be fostering relationships as part of their journey out.

The trend analysis also revealed that the gender difference in the number of homeless individuals has become greater over the three survey years. This is in line with the literature, where males predominantly represent the homeless population compared to their female counterparts [9, 12, 14, 22–25].

The age of respondents produced a particularly interesting finding, where older individuals were found to experience first-time homelessness more frequently since the beginning of the COVID-19 pandemic. This finding suggests that those over the age of 25 were particularly susceptible to COVID-related housing loss. This abrupt social change acted as an adverse life event for the “potentially homeless” age group, propelling them into a life of homelessness. This could be due to several reasons directly caused by the pandemic including, but not limited to reduced accessibility to supports [22], and loss of employment [26].

The location where homeless individuals sleep also appeared to be influenced by the pandemic. In 2020, availability of emergency shelters, institutional settings, and places where individuals could “couch surf” decreased. However, the number of individuals staying in hotels/motels funded by a homeless program, or in unsheltered/unknown locations increased. These findings can be attributed to reduced capacities of emergency shelters and inability to socially distance there during the COVID-19 pandemic. The hotel/motel room rentals were an active and effective temporary response to these dilemmas. The Centre for Disease Control and Prevention recommended this solution to service and support providers across Canada to continue service delivery while maintaining the required safety protocols [27]. The ability to separate individuals and/or families into their own hotel rooms allowed for both social distancing and extra beds while emergency shelters operated with reduced client capacities. Examples of these hotel placements could be seen across Ontario and Québec. This solution can still be seen in operation in North Bay, Ontario. It becomes clear with these results that the COVID-19 pandemic not only affected homelessness trends on an individual level, but on the macro level.

Another interesting result was that chronic homelessness, defined as individuals experienced homelessness for 180 days or more of a given 12-month period [28], has increased over the last three survey years. When combined with the finding that respondents were found to experience fewer episodes of homelessness in the same period, the results suggest that individuals are becoming homeless and continuously staying homeless for prolonged periods of time. This notion was supported by Chamberlain and Johnson [29], who argued that individuals experiencing homelessness become accustomed to the lifestyle through the process of social adaptation. This in turn escalates the difficulty to “rise out” further leading to a state of chronicity. One of the reasons for the prolonged period of homelessness can be the likely perpetual adverse social conditions, leading to homelessness, and constant engagement in “maladaptive lifestyles”, such as using drugs or excessive alcohol drinking, and being in trouble with the law [30]. Moreover, specific barriers to housing were noted to have significantly increased since the beginning of the pandemic including addiction and substance use, and the inaccessibility to safe and secure rental units, perhaps due to the drastic spike in housing markets observed over the last year [31].

### Policy recommendations

The results of this research have policy implications for homelessness in the Nipissing District. First, the findings highlighted certain population groups that need a targeted program to address their needs. It is therefore necessary, for the development and implementation of a Coordinated Access service system, as indicated on DNSSAB’s website, to continue [19]. This will allow for collaborative service-delivery methods, perhaps resulting in matching clients to appropriate resources more efficiently. It is evident that those under the age of 25 are vulnerable to experiencing homelessness. As such, an increase in the supports available to youth clients in the District is required as a first step towards ending youth homelessness. The findings also necessitate the recommendation for service and support agencies to advocate for increased welfare allocations to support inflation of living expenses. Previous research has documented that individuals who spend 30% or more of their annual income on living expenses are susceptible to losing their housing [5]. The results of the 2021 survey saw a dramatic increase in the inaccessibility of rental units, making it clear that these supports are not meeting client needs.

### Limitations

Although the findings of this study could be considered the most reliable analyses of homelessness in the District of Nipissing to date, this study has limitations that may be addressed in future studies. First, the data gathered from close-ended enumeration survey questions are not so rich and in-depth that one can interpret the rationales behind the complex process of homelessness. Therefore, research with a qualitative approach could perhaps have accomplished this goal. Second, not all enumerated homeless individuals were willing to participate in the enumeration survey interviews. Since there were no background information about non-respondents, it was not possible to evaluate for any likely selection biases in the findings. Third, the cross-sectional data collected by the enumeration surveys are limited in examining the casual effect of pandemic on homelessness, as there could be many other unknown factors, in addition to the pandemic, that could be co-occurring and affecting homelessness and the demographic composition of homeless individuals. Lastly, the number of enumerated individuals that experienced COVID-related housing loss was relatively small for extended analyses. A future study focusing specifically on this experience is recommended.

## Conclusion

Based on the trend analysis, gender disparity in the homeless population in the Nipissing District and first-time homelessness among middle-aged and older individuals are growing. Moreover, the results revealed a decrease in the use of emergency shelters, transitional housing, and institutional resources, but an increase in hotel/motel rentals because of the pandemic. An increase in interpersonal/family issues directly causing homelessness was observed, as well as an increase in chronicity despite a decrease in episodic homelessness. Significant increases in addiction-based barriers to housing were revealed, along with the inaccessibility of rental units preventing individuals from securing safe and affordable housing options, and further prolonging the duration of their homeless periods. The results of this study suggest that in developing new homelessness programs and policies, or in tailoring existing ones, policy makers in the District of Nipissing need to focus on increasing homeless supports and preventative measures for identified at-risk individuals, and advocate for increased welfare allocations to support inflation of living expenses.

## Electronic supplementary material

Below is the link to the electronic supplementary material.

**Appendix A:** 2021 Enumeration Survey Questionnaire, Nipissing District, Ontario, Canada.

## Data Availability

The data analysed during the current study are publicly available for 2018 and 2020 on the webpage of District of Nipissing Social Services Administration Board (DNSSAB): https://www.dnssab.ca, and for the 2021 microdata set, a request should be made to DNSSAB.
