# Supplementary material for: Homelessness in the district of Nipissing of Ontario, Canada before, at the onset and during the COVID-19 pandemic: a trend analysis (2018–2021)
Source: BMC Public Health. 2023 Jul 19;23:1388. doi: 10.1186/s12889-023-16314-2 (PMC10354900; doi:10.1186/s12889-023-16314-2)
Supplement: Supplementary file 1 — Appendix A: 2021 Enumeration Survey Questionnaire, Nipissing District, Ontario, Canada. [file 12889_2023_16314_MOESM1_ESM.pdf]

**NIPISSING COUNTS 2021 SURVEY** **SURVEY #**

**Location**: ________________________________ **Date (mm/dd) / Time**: _______________ (INDICATE AM/PM)

**Interviewer Name**: ______________________________________________________________________

**Include this survey in count:**

| | |
|---|---|
| YES | NO |

ONLY INCLUDE THIS SURVEY IN THE COUNT, IF THE INDIVIDUAL WAS HOMELESS ON WEDNESDAY OCTOBER 13TH. IF NOT, THIS SURVEY IS TO BE OMITTED FROM THE COUNT, AND USED FOR THE BNL.

## OPENING SCRIPT

**In which language do you feel best able to express yourself?**

| | | |
|---|---|---|
| ☐ ENGLISH<br>☐ FRENCH (see French survey attached)<br>☐ CREE (call 705-491-6593 to access Cree translator) | ☐ NO PREFERENCE<br>☐ INDIGENOUS LANGUAGE NOT LISTED _______________________<br>☐ OJIBWAY (call 705-491-6593 to access Ojibway translator) | ☐ DON'T KNOW<br>☐ DECLINE TO ANSWER<br>☐ NOT LISTED (please specify) ___________________________ |

Hello, my name is ________________ and I am a surveyor with Nipissing Counts 2021. Today, we are surveying individuals who may be homeless and/or couch surfing. This survey takes about 10 minutes to complete.

- Your participation is voluntary, and your name will not be recorded without your consent.
- You can choose to skip any questions or to stop the interview at any time.
- The results from this survey will help us better understand homelessness in our communities
- Your responses will not affect your relationship with the people and agencies supporting you.

This year, we are also building a By-Name List registry that will allow different agencies to work together, to better service you. By adding yourself to the registry, it will save you from having to repeat information any time you access services across our District. Please note that any identifying information will only be accessible to agencies working with you and will not be accessible to any provincial or federal government agencies. Should you wish to keep your name anonymous, a non-identifying marker will be created for you.

**With this information in mind, would you like the information collected through this survey to be used to add you to the By-Name List Registry?**

| | |
|---|---|
| YES | NO |

IF YES:

Thank you for agreeing to be part of this initiative. Within TIME PERIOD a front-line worker will reach out to you, to ask some more questions so that we may best respond to your needs.

## SCREENING QUESTIONS

Have you answered this survey with another person wearing this badge?

| | |
|---|---|
| YES | Please continue the survey. Go to next question. |
| NO | END SURVEY. THANK YOU FOR YOUR PARTICIPATION & TALLY THE INDIVIDUAL. |

Are you willing to participate in the survey?

| | |
|---|---|
| YES | Please continue the survey. Go to next question. |
| NO | END SURVEY. THANK YOU FOR YOUR PARTICIPATION & TALLY THE INDIVIDUAL. |

Has informed, verbal consent been received to administer this survey?

| | |
|---|---|
| YES | Please continue the survey. Go to next question. |
| NO | END SURVEY. THANK YOU FOR YOUR PARTICIPATION & TALLY THE INDIVIDUAL. |

Where are you staying tonight?

| | |
|---|---|
| ☐ OWN APARTMENT/HOUSE/ROOM RENTAL/MONTHLY MOTEL/HOTEL RENTAL<br>☐ SOMEONE ELSE'S PLACE (COUCH SURFING)<br>☐ HOSPITAL<br>☐ JAIL, PRISON, REMAND CENTRE, DETENTION CENTRE<br>☐ MOTEL/HOTEL (SELF-FUNDED ON DAILY BASIS)<br>☐ TREATMENT CENTRE/DETOX<br><br>☐ UNSURE: IF SCREENED IN THEN INDICATE PROBABLY LOCATION ON NEXT PAGE | Do you have an apartment, room or house of your own where you can return to and/or stay as long as you want?<br><br>YES [THANK AND END SURVEY]<br>NO [CONTINUE]<br>DON'T KNOW [CONTINUE]<br>DECLINE TO ANSWER [THANK AND END SURVEY] |
| ☐ HOMELESS SHELTER (EMERGENCY, FAMILY OR DOMESTIC VIOLENCE SHELTER)<br>☐ WARMING CENTRE<br>☐ HOTEL/MOTEL (FUNDED BY A HOMELESS PROGRAM)<br>☐ TRANSITIONAL SHELTER/HOUSING<br>☐ PUBLIC SPACE (IE. STREET, PARK, BUS SHELTER, FOREST, ENCAMPMENT)<br>☐ ABANDONED/VACANT BUILDING<br>☐ C-CAN, STORAGE CONTAINER, STORAGE UNIT<br>☐ VEHICLE (CAR, VAN, RV, MOTOR HOME, NON-PERMANENT CAMPING TRAILER, TRUCK, BOAT)<br>☐ OTHER UNSHELTERED LOCATION [WRITE RESPONSE ON NEXT PAGE] | CONTINUE |

| NIPISSING COUNTS 2021 SURVEY | SURVEY # |
|---|---|

## RESPONDENT CODED ID

**CODED I.D:** _______ _______ _______ _______

FIRST TWO LETTERS OF FIRST NAME — FIRST TWO LETTERS OF LAST NAME

## RESPONDENT INFORMATION

1. What is your first and last name? FOR USE ON BY-NAME LIST ONLY - SKIP IF RESPONDENT WISHES TO REMAIN ANONYMOUS

| | |
|---|---|
| Last Name | |
| First Name | |
| Nickname(s) | |

2. What is the best way to follow up with you?

| | |
|---|---|
| PHONE: | EMAIL: |
| OTHER: | |

3. How old are you? [OR] What year were you born? [If unsure, ask for an estimate]

| | | |
|---|---|---|
| AGE ______ OR YEAR BORN ___ ___ ___ ___ | ☐ DON'T KNOW | ☐ DECLINE TO ANSWER |

4. Which gender do you identify with? [Show or read list]

| | | |
|---|---|---|
| ☐ MALE<br>☐ FEMALE<br>☐ TWO-SPIRIT | ☐ MALE-TO-FEMALE TRANS<br>☐ FEMALE-TO-MALE TRANS<br>☐ NON-BINARY | ☐ DON'T KNOW<br>☐ DECLINE TO ANSWER<br>☐ NOT LISTED: ____________________ |

5. How would you describe your sexual orientation, for example straight, gay, lesbian? [Show or read list]

| | | | |
|---|---|---|---|
| ☐ STRAIGHT<br>☐ GAY<br>☐ LESBIAN | ☐ BISEXUAL<br>☐ TWO-SPIRIT<br>☐ PANSEXUAL | ☐ ASEXUAL<br>☐ QUEER<br>☐ QUESTIONING/UNSURE | ☐ DECLINE TO ANSWER<br>☐ NOT LISTED: ____________________ |

6. Which of the following best describes your citizenship/immigrant status?

| | |
|---|---|
| ☐ CANADIAN CITIZEN (BORN IN CANADA)<br>☐ CANADIAN CITIZEN (BORN OUTSIDE OF CANADA)<br>☐ PERMANENT RESIDENT/IMMIGRANT<br>☐ REFUGEE<br>☐ REFUGEE CLAIMANT | ☐ STUDENT VISA<br>☐ VISITOR VISA<br>☐ WORK VISA<br>☐ UNDELCARED |
| IF YOU SELECTED ANYTHING OTHER THAN "CANADIAN CITIZEN (BORN IN CANADA)" PLEASE SPECIFY YOUR COUNTRY OF BIRTH BELOW: | |
| COUNTRY OF BIRTH: | |

IF THE RESPONDENT ANSWERED "CANADIAN CITIZEN (BORN IN CANADA)", SKIP TO QUESTION 8.

7. How long have you been in Canada?

| | |
|---|---|
| LENGTH _____________ DAYS / WEEKS / MONTHS / YEARS | ☐ DON'T KNOW<br>☐ DECLINE TO ANSWER |

8. Do you identify as First Nations, Métis, or Inuit, or do you have North American Indigenous ancestry?

| | | |
|---|---|---|
| ☐ YES<br>☐ NO<br>☐ DON'T KNOW<br>☐ DECLINE TO ANSWER | IF YES: | ☐ FIRST NATIONS (with or without status)<br>☐ INUIT<br>☐ MÉTIS<br>☐ SELF IDENTIFY |
| | IF YES, WHICH INDIEGNOUS COMMUNITY/BAND ARE YOU FROM?<br>☐ COMMUNITY/BAND NAME: ________________________<br>☐ DON'T KNOW<br>☐ DECLINE TO ANSWER | |

IF THE RESPONDENT ANSWERED "YES" TO QUESTION 8, SELECT "INDIGENOUS" AND MOVE TO QUESTION 10.

9. What ancestry do you identify with?

| | |
|---|---|
| ☐ INDIGENOUS<br>☐ ARAB<br>☐ ASIAN (IE. CHINESE, KOREAN, JAPANESE)<br>☐ BLACK OR AFRICAN CANADIAN<br>☐ FILIPINO<br>☐ HISPANIC OR LATIN AMERICAN<br>☐ SOUTH ASIAN (EAST INDIAN, PAKISTANI, SRI LANKAN)<br>☐ SOUTH-EAST ASIAN (VIETNAMESE, CAMBODIAN, MALAYSIAN, LAOTIAN) | ☐ WEST ASIAN (IRANIAN, AFGHAN)<br>☐ WHITE (EUROPEAN-CANADIAN)<br>☐ DON'T KNOW<br>☐ DECLINE TO ANSWER<br>☐ OTHER (PLEASE SPECIFY):<br>___________________________________ |

10. Have you ever served in the Canadian Military or Royal Canadian Mounted Police (RCMP)? (Military includes Canadian Navy, Army or Air Force)

| | | |
|---|---|---|
| ☐ YES, MILITARY<br>☐ YES, RCMP | ☐ BOTH MILITARY & RCMP<br>☐ NO | ☐ DON'T KNOW<br>☐ DECLINE TO ANSWER |

11. Are you currently a student enrolled at a university or college?

| | |
|---|---|
| ☐ YES | ☐ NO |

12. How would you rate your overall health?

| | | | | |
|---|---|---|---|---|
| ☐ VERY GOOD | ☐ GOOD | ☐ FAIR | ☐ POOR | ☐ VERY POOR |
| ☐ DON'T KNOW | | ☐ DECLINE TO ANSWER | | |

13. Do you experience challenges related to any of the following:

| | | | | |
|---|---|---|---|---|
| ILLNESS OR MEDICAL CONDITION (ie. Diabetes, arthritis, TB, HIV) | ☐ YES | ☐ NO | ☐ DON'T KNOW | ☐ DECLINE TO ANSWER |
| PHYSICAL DISABILITY (ie. Mobility issues, dexterity, capacity) | ☐ YES | ☐ NO | ☐ DON'T KNOW | ☐ DECLINE TO ANSWER |
| LEARNING DISABILITY (ie. ADHD, dyslexia, autism spectrum disorder, brain injury) | ☐ YES | ☐ NO | ☐ DON'T KNOW | ☐ DECLINE TO ANSWER |
| MENTAL HEALTH (ie. depression, anxiety, PTSD, bipolar disorder) | ☐ YES | ☐ NO | ☐ DON'T KNOW | ☐ DECLINE TO ANSWER |
| SUBSTANCE ABUSE (ie. tobacco, alcohol, opiates) | ☐ YES | ☐ NO | ☐ DON'T KNOW | ☐ DECLINE TO ANSWER |

IF THE RESPONDENT ANSWERED "YES" TO "SUBSTANCE ABUSE" ABOVE, ASK Q14, IF NOT CONTINUE TO Q15.

14. If you were offered a place in a treatment program today, would you accept it?

| | |
|---|---|
| ☐ YES | ☐ NO |

15. Do you have any family members or anyone else who is staying with you tonight?

| | |
|---|---|
| ☐ NONE<br>☐ PARTNER – survey # ___ ___ ___ ___<br>☐ PARTNER - survey # unknown | ☐ OTHER ADULT(S) (can include other family or friends)<br>☐ DECLINE TO ANSWER |

| ☐ CHILDREN/DEPENDENTS | | 1 | 2 | 3 | 4 | 5 | 6 | 7 |
|---|---|---|---|---|---|---|---|---|
| Please indicate the gender identity and age for each. | GENDER | | | | | | | |
| | AGE | | | | | | | |

## HOMELESSNESS HISTORY

FOR THIS SURVEY, THE TERM "HOMELESSNESS" REFERS TO ANY TIME WHEN YOU HAVE BEEN WITHOUT A PERMANENT AND SECURE PLACE TO LIVE, INCLUDING SLEEPING IN SHELTERS, ON THE STREETS, OR LIVING TEMPORARILY WITH OTHERS WITHOUT HAVING YOUR OWN PERMANENT HOUSING (IE. COUCH SURFING).

16. **How old were you the first time you experienced homelessness?**

| | | |
|---|---|---|
| AGE: ____________ | ☐ DON'T KNOW | ☐ DECLINE TO ANSWER |

17. **What happened that caused you to lose your housing for the first time?** [Do not read the options] Please **check all that apply**. "Housing" does not include temporary arrangements (ie. couch surfing) or shelter stays. Follow up for specific reason if the respondent says "eviction" or that they "chose to leave".

| HOUSING AND FINANCIAL LOSS | INTERPERSONAL AND FAMILY ISSUES | OTHER |
|---|---|---|
| ☐ BUILDING SOLD OR RENNOVATED<br>☐ COMPLAINT (IE. PETS, NOISE, DAMAGE)<br>☐ LEFT COMMUNITY/RELOCATED<br>☐ NOT ENOUGH INCOME FOR HOUSING<br>☐ OWNER MOVED IN<br>☐ UNFIT/UNSAFE HOUSING CONDITION<br>**HEALTH RELATED**<br>☐ HOSPITALIZATION OR TREATMENT PROGRAM<br>☐ MENTAL HEALTH<br>☐ PHYSICAL HEALTH<br>☐ SUBSTANCE ABUSE | ☐ EXPERIENCED STIGMA/DISCRIMINATION<br>☐ CONFLICT WITH TENNANT/LANDLORD<br>☐ CONFLICT WITH PARENT/GUARDIAN<br>☐ CONFLICT WITH SPOUSE/PARTNER<br>☐ CONFLICT WITH OTHER<br>☐ DEATH OF FAMILY MEMBER<br>☐ DEPARTURE OF FAMILY MEMBER<br>☐ EXPERIENCED ABUSE FROM PARENT/GUARDIAN<br>☐ EXPERIENCED ABUSE FROM SPOUSE/PARTNER<br>☐ EXPERIENCED ABUSE BY OTHER<br>☐ FAMILY BREAKDOWN<br>☐ RELATED TO FOSTER CARE/ADOPTION | ☐ INCARCERATION (JAIL OR PRISON)<br>☐ OTHER (please specify) ________________________<br>☐ DON'T KNOW<br>☐ DECLINE TO ANSWER |

18. **In total, how many <u>different times</u> have you experiences homelessness over the last 12 months?**

| | | |
|---|---|---|
| NUMBER OF TIMES: ____________ (including this time) | ☐ DON'T KNOW | ☐ DECLINE TO ANSWER |

19. **In total, how <u>much time</u> have you been homeless or couch surfing over the last 12 months?**

| | | |
|---|---|---|
| LENGTH ____________ DAYS / WEEKS / MONTHS | ☐ DON'T KNOW | ☐ DECLINE TO ANSWER |

20. **How long have you been in LOCATION?**

| | |
|---|---|
| ☐ LENGTH _______ DAYS / WEEKS / MONTHS / YEARS<br>☐ ALWAYS BEEN HERE (skip to Q22)<br>☐ DON'T KNOW (skip to Q22)<br>☐ DECLINE TO ANSWER (skip to Q22) | Where did you live before coming here?<br>_________________________________________<br>(COMMUNITY, PROVINCE AND/OR COUNTRY)<br>☐ DECLINE TO ANSWER |

21. **What is the main reason you came to LOCATION?** [Do not read categories – **select only one**]

| | | |
|---|---|---|
| ☐ EMPLOYMENT - SECURED<br>☐ EMPLOYMENT – SEEKING<br>☐ ENVIRONMENTAL DISPLACEMENT (ie. water, flooding, mould, fire, etc.)<br>☐ FAMILY MOVED HERE<br>☐ FEAR FOR PERSONAL SAFETY<br>☐ RECREATION / SHOPPING | ☐ TO ATTEND SCHOOL<br>☐ TO ACCESS EMERGENCY SHELTER(S)<br>☐ TO ACCESS SERVICES AND SUPPORTS<br>☐ TO FIND HOUSING<br>☐ TO VISIT FRIENDS/FAMILY | ☐ DON'T KNOW<br>☐ DECLINE TO ANSWER<br>☐ OTHER: ________________________ |

22. **What happened that caused you to lose your housing most recently?** [Do not read the options. **Check all that apply**. "Housing" does not include temporary arrangements or shelter stays. Follow up if the respondent says "eviction" or that they "chose to leave"]

| HOUSING AND FINANCIAL LOSS | INTERPERSONAL AND FAMILY ISSUES | OTHER |
|---|---|---|
| ☐ BUILDING SOLD OR RENNOVATED | ☐ EXPERIENCED STIGMA/DISCRIMINATION | ☐ INCARCERATION (JAIL OR PRISON) |
| ☐ COMPLAINT (IE. PETS, NOISE, DAMAGE) | ☐ CONFLICT WITH TENNANT/LANDLORD | ☐ OTHER (please specify) ________________________ |
| ☐ LEFT COMMUNITY/RELOCATED | ☐ CONFLICT WITH PARENT/GUARDIAN | ☐ DON'T KNOW |
| ☐ NOT ENOUGH INCOME FOR HOUSING | ☐ CONFLICT WITH SPOUSE/PARTNER | ☐ DECLINE TO ANSWER |
| ☐ OWNER MOVED IN | ☐ CONFLICT WITH OTHER | |
| ☐ UNFIT/UNSAFE HOUSING CONDITION | ☐ DEATH OF FAMILY MEMBER | |
| **HEALTH RELATED** | ☐ DEPARTURE OF FAMILY MEMBER | |
| ☐ HOSPITALIZATION OR TREATMENT PROGRAM | ☐ EXPERIENCED ABUSE FROM PARENT/GUARDIAN | |
| ☐ MENTAL HEALTH | ☐ EXPERIENCED ABUSE FROM SPOUSE/PARTNER | |
| ☐ PHYSICAL HEALTH | ☐ EXPERIENCED ABUSE BY OTHER | |
| ☐ SUBSTANCE ABUSE | ☐ FAMILY BREAKDOWN | |
| | ☐ RELATED TO FOSTER CARE/ADOPTION | |

23. **Was your most recent housing loss related to the COVID-19 pandemic?**

| | | | |
|---|---|---|---|
| ☐ YES | ☐ NO | ☐ DON'T KNOW | ☐ DECLINE TO ANSWER |

24. **How long ago did that happen (that you most recently lost housing)?** [Best estimate]

| | | |
|---|---|---|
| LENGTH _____________ DAYS / WEEKS / MONTHS | ☐ DON'T KNOW | ☐ DECLINE TO ANSWER |

25. **What challenges or problems have you experienced when trying to find housing?** [Please check all that apply]

| | | |
|---|---|---|
| ☐ ADDICTION | ☐ POOR MENTAL HEALTH | ☐ DON'T WANT HOUSING |
| ☐ SUBSTANCE USE | ☐ MENTAL ILLNESS | ☐ NO BARRIERS TO HOUSING |
| ☐ BAD CREDIT | ☐ NO INCOME ASSISTANCE | ☐ NONE OF THE ABOVE |
| ☐ CHILDREN | ☐ NO REFERENCES | ☐ DECLINE TO ANSWER |
| ☐ CRIMINAL HISTORY | ☐ PETS | ☐ OTHER: ____________________________ |
| ☐ DOMESTIC VIOLENCE | ☐ POOR HOUSING CONDITIONS | |
| ☐ FAMILY BREAKDOWN / CONFLICT | ☐ RACISM | |
| ☐ POOR PHYSICAL HEALTH | ☐ RENTAL UNITS NOT AVAILABLE | |
| ☐ DISABILITY | ☐ RENT IS TOO HIGH | |
| ☐ LACK OF APPROPRIATE UNIT SIZE | ☐ STIGMA / DISCRIMINATION | |
| ☐ LOW INCOME | | |

26. **What are you plans for winter with regards to housing?**

| | |
|---|---|
| ☐ STAYING IN THE COMMUNITY: | ☐ LEAVING THE COMMUNITY |
| ☐ STAYING WITH FRIENDS/FAMILY | ☐ DON'T KNOW |
| ☐ EMERGENCY SHELTERS | ☐ DECLINE TO ANSWER |
| ☐ HOTEL/MOTEL RENTAL | ☐ OTHER: __________________________ |
| ☐ LIVING UNSHELTERED | |

27. **What are your sources of income?** [Read list and check all that apply]

| | | |
|---|---|---|
| ☐ BAND FUNDING | ☐ INFORMAL INCOME SOURCES (IE. BOTTLE RETURNS, PANHANDLING) | ☐ VETERAN / VAC BENEFITS |
| ☐ CAS BENEFIT | ☐ MONEY FROM FAMILY / FRIENDS | ☐ WSIB |
| ☐ CASUAL EMPLOYMENT (IE. CONTRACT WORK) | ☐ ONTARIO DISABILITY SUPPORT PROGRAM (ODSP) | ☐ OTHER MONEY FROM A SERVICE AGENCY |
| ☐ CHILD AND FAMILY TAX BENEFITS (ie. Baby Bonus) | ☐ ONTARIO WORKS | ☐ NO INCOME |
| ☐ CORRECTION SERVICES CANADA (CSC BENEFIT) | ☐ OSAP | ☐ DON'T KNOW |
| ☐ CPP-DISABILITY | ☐ PRIVATE PENSION | ☐ DECLINE TO ANSWER |
| ☐ EMPLOYMENT INSURANCE (EI) | ☐ SENIORS BENEFITS (IE. CPP/OAS/GIS) | ☐ OTHER SOURCE: _________________________ |
| ☐ EMPLOYMENT (FULL-TIME) | ☐ SPOUSAL SUPPORT | |
| ☐ EMPLOYMENT (PART-TIME) | ☐ TRILIUM | |
| ☐ GST/HST REFUND | | |

28. **In the past year (12 months) have you:** [Ask respondents to give best estimate]

| | | | | |
|---|---|---|---|---|
| BEEN TO A HOSPITAL EMERGENCY ROOM | Y _____ N _____ | # OF TIMES __________ | ☐ DON'T KNOW | ☐ DECLINE TO ANSWER |
| BEEN HOSPITALIZED<br><br>DAYS YOU SPENT HOSPITALIZED | Y _____ N _____ | # OF TIMES __________<br><br>DAYS SPENT __________ | ☐ DON'T KNOW<br><br>☐ DON'T KNOW | ☐ DECLINE TO ANSWER<br><br>☐ DECLINE TO ANSWER |
| INTERACTED WITH POLICE<br>(ie. tickets, arrests, searches) | Y _____ N _____ | # OF TIMES __________ | ☐ DON'T KNOW | ☐ DECLINE TO ANSWER |
| BEEN TO JAIL / PRISON<br><br>DAYS YOU SPENT IN JAIL / PRISON | Y _____ N _____ | # OF TIMES __________<br><br>DAYS SPENT __________ | ☐ DON'T KNOW<br><br>☐ DON'T KNOW | ☐ DECLINE TO ANSWER<br><br>☐ DECLINE TO ANSWER |

29. **As a child or youth, were you ever in foster care or in a youth group home?**

| | | | |
|---|---|---|---|
| ☐ YES | ☐ NO | ☐ DON'T KNOW | ☐ DECLINE TO ANSWER |

## WRAPPING UP THE SURVEY

If this survey has brought up any strong feelings, and you are interested in supports located in the community, please know that inside this honorarium is a 211 card and a pamphlet about local services. You can call 211 from any phone and an operator will connect you with services that can support you.

Thank you for your time and for generously sharing your story. Your input and information is very valuable, and will help us better understand homelessness in our community and the needs of those experiencing it.

Do you have any questions?
